# Inhibitory Effect of Glutathione on Oxidative Liver Injury Induced by Dengue Virus Serotype 2 Infections in Mice

**DOI:** 10.1371/journal.pone.0055407

**Published:** 2013-01-30

**Authors:** Juan Wang, Yanlei Chen, Na Gao, Yisong Wang, Yanping Tian, Jiangman Wu, Junlei Zhang, Junping Zhu, Dongying Fan, Jing An

**Affiliations:** 1 Department of Microbiology, School of Basic Medical Sciences, Capital Medical University, Beijing, People’s Republic of China; 2 Department of Histology and Embryology, Microbiology, Third Military Medical University, Chongqing, People’s Republic of China; University of Rochester, United States of America

## Abstract

The pathogenesis of dengue virus (DV) infection has not been completely defined and change of redox status mediated by depletion of glutathione (GSH) in host cell is a common result of viral infection. Our previous study has demonstrated that DV serotype 2 (DV2) infection alters host intracellular GSH levels, and exogenous GSH inhibits viral production by modulating the activity of NF-κB in HepG2 cells. GSH is the most powerful intracellular antioxidant and involved in viral infections. Thus, this study was to investigate whether DV2 infection can induce alteration in redox balance and effect of GSH on the disease in HepG2 xenografts SCID mice. Our results revealed that mice infected with DV2 showed alterations in oxidative stress by increasing the level of malondialdehyde (MDA), an end product of lipid peroxidation, and GSSG/GSH ratio. DV2-infected mice also showed a decrease in the activity of catalase (CAT) and total superoxide dismutase (T-SOD) in the serum and/or observed organs, especially the liver. Moreover, DV2 infection resulted in elevated serum levels of the cytokines tumor necrosis factor-α and interlukin-6 and obvious histopathological changes in the liver. The administration of exogenous GSH significantly reversed all of the aforementioned pathological changes and prevented significant liver damage. Furthermore, in vitro treatment of HepG2 cells with antioxidants such as GSH inhibited viral entry as well as the production of reactive oxygen species in HepG2 cells. These results suggest that GSH prevents DV2-induced oxidative stress and liver injury in mice by inhibiting proinflammatory cytokine production, and GSH and may be a promising therapeutic agent for prevention of oxidative liver damage during DV infection.

## Introduction

Dengue virus (DV) is a member of the family *Flaviviridae* and is one of the most widespread mosquito-borne human pathogens worldwide. There are four antigenically distinct serotypes (DV1–4) based on differences in the envelope protein. DV is the causative agent of dengue fever (DF), a mild, self-limiting disease that typically presents with high fever, severe headache, and pain behind the eyes. A minority of patients may progress to life-threatening dengue hemorrhagic fever (DHF) and dengue shock syndrome (DSS) characterized by systemic hemorrhage and increased capillary permeability. DHF and DSS are often associated with an enlarged liver, jaundice, increased levels of transaminases and prolonged partial thromboplastin times, which indicate liver dysfunction and injury [1]–[5]. Moreover, the proliferation of DV has been detected in vitro with apparent cytopathic effects (CPE) in different liver cell lines such as the human hepatocarcinoma cell line HepG2 [6]. Hepatocytes can support DV replication, and the liver is thought to be an important target organ for DV infection.

The pathogenesis of DHF/DSS with respect to liver injury remains poorly understood. The replication of DV depends on numerous host cellular factors that control various cellular processes involved in cellular metabolism and contribute to the pathogenesis of DV infection. Recently, the intracellular redox balance was proposed to be involved in viral infections and the progression of viral diseases. Increased reactive oxygen species (ROS) cause a significant proportion of the damage to virus-infected cells, and ROS can be neutralized by antioxidant molecules such as glutathione (GSH), superoxide dismutase (SOD), thioredoxin (Trx), and catalase (CAT), which constitute the cellular system that counteracts oxidation and play important roles in maintaining a reductive intracellular environment. GSH is a cysteine-containing tripeptide that is the most important and ubiquitous antioxidant molecule produced in human organs. GSH is particularly important in the liver where it serves as the principal non-protein thiol involved in the cellular antioxidant defense.

Previous studies have demonstrated that cultured cells infected with herpes simplex virus type 1 (HSV-1) [7], Sendai virus [8], and human immunodeficiency virus (HIV) [9] show reduced levels of intracellular GSH, increased generation of ROS and oxidation of the cellular GSH pool. This leads to the activation of redox-dependent transcription factors such as NF-κB and leads to the increased production of various cytokines including tumor necrosis factor-α (TNF-α) and interlukin-6 (IL-6). A decrease in the level of GSH has also been reported in patients infected with both DNA and RNA viruses [10], [11]. In patients with HIV infection, low GSH levels were found in plasma, erythrocytes, T cells and monocytes. Some antioxidant molecules such as GSH, glutathione ester and *N*-acetyl-l-cysteine (NAC, a precursor of GSH) were able to suppress HIV replication in chronically infected monocytic cells [12]. GSH might interfere with the entry of some viruses such as HIV and rhinovirus by changing the intracellular redox status [13]. Previous studies have shown that GSH exerts an inhibitory effect on HIV and influenza A virus infections in cultured cells [14] and animals [15], [16]. These results suggest that the change in the intracellular redox status is influenced by the depletion of GSH, which commonly occurs as a result of viral infection. Furthermore, the change in redox status may vary in intensity, duration and mechanism of induction, depending on the type of virus.

More recently, oxidative damage was observed in patients with DV infections, suggesting that oxidative stress could also play an important role in the pathogenesis of DF or DHF/DSS [17], [18]. We have recently reported that DV2 infection alters the host intracellular GSH levels, and exogenous GSH inhibits viral production by modulating the activity of NF-κB in HepG2 cells. In contrast, buthionine sulfoximine (BSO), an inhibitor of GSH synthesis, effectively enhanced DV2 replication in HepG2 cells [19], indicating a general relationship between GSH and viral replication and implicating GSH as a potential therapeutic agent for the treatment of viral infections via the inhibition of oxidative stress. However, whether GSH is effective on DV2 infection in vivo remains unknown.

In the present study, HepG2-grafted severe combined immunodeficiency disease (HepG2-grafted SCID) mice were used to investigate the inhibitory effect of GSH on oxidative stress induced by DV2 infection. Changes in the intracellular redox status and oxidative stress injury were evaluated by measuring the levels of malondialdehyde (MDA, an end product of lipid peroxidation), the GSSG/GSH ratio, the activities of anti-oxidative enzymes (AOEs) such as SOD and CAT in the serum and/or organs and the serum levels of the inflammatory cytokines TNF-α and IL-6. Histopathological examination was also performed on major organs of the infected mice. Furthermore, the inhibitory effect of antioxidants including GSH, NAC and vitamin C (Vit C) on the aforementioned parameters, viral replication and ROS production was investigated using HepG2-grafted SCID mice and HepG2 cells. Our results showed that DV2 infection could cause oxidative stress both in vivo and in vitro. Supplemental GSH could inhibit oxidative stress, effectively reducing oxidative damage to the organs, especially the liver. Our results suggest that GSH may be a useful agent in the prevention and treatment of liver damage elicited by lipid peroxidation during DV infection.

## Materials and Methods

### Ethics Statement

This study was carried out in strict accordance with the recommendations in the national guidelines for the use of animals in scientific research “Regulations for the Administration of Affairs Concerning Experimental Animals”. The protocol was also approved by the Animal Care and Use Committee of Chinese Capital Medical University (Permit Number 2009-X-871). All surgeries were performed under diethyl ether anesthesia, and all efforts were made to minimize suffering.

### Cell Culture, Virus and Reagents

Vero cells were cultured in minimum essential medium (Gibco) supplemented with 5% fetal bovine serum (FBS). HepG2 cells were cultured in Dulbecco’s Modified Eagle Medium (DMEM, Gibco) supplemented with 10% FBS. C6/36 cells were cultured in RPMI1640 (Gibco) supplemented with 10% FBS.

DV2 (strain Tr1751), isolated from a patient with dengue fever, was propagated in C6/36 cells and stored at −80°C until use. The viral titer was detected by a plaque assay that used a Vero cell monolayer cultured with 1.3% methylcellulose overlay medium.

The antioxidants L-glutathione (GSH), N-Acetyl-L-cysteine (NAC), ascorbic acid (Vitamin C, Vit C) and buthionine sulfoximine (BSO) were purchased from Sigma-Aldrich and were dissolved in 0.9% NaCl solution (pH 7.4).

### Mouse Model for DV2 Infection

Six-week-old female SCID mice were purchased from Vital River Laboratories (Beijing, China) and were housed in a pathogen-free environment supplied with sterile water and food. The previously described HepG2 xenograft SCID mouse model (HepG2-SCID) was used in this study with some modifications [20]. In brief, the mice were injected intraperitoneally (i.p.) with 5×10^6^ HepG2 cells. The concentration of hALB in the serum was monitored at various time points post-transplantation (p.t.) by using an enzyme-linked immunosorbent assay (ELISA) to confirm successful transplantation. Mice were challenged i.p. with DV2 (1×10^6^ PFU) on day 10 p.t. and were then divided into two groups. One group of mice was evaluated for evidence of disease progression, and the other group was sacrificed at day eight post-infection (p.i.) for virus detection in the serum and organs, as well as for histopathological examination.

### Experimental Design: Infection, Antioxidant Administration and Sample Collection

The effect of various antioxidants on DV2 infection in vivo was performed using the HepG2-SCID model. Mice were divided into eight groups with seven mice in each group. Mice that were treated with 1, 3 and 8 mg/mouse per day of GSH were referred to as groups G1, G3 and G8, respectively. Similarly, mice that were treated with 1 and 2 mg/mouse per day of NAC were referred to as groups N1 and N2, respectively. Mice that were treated with 1 mg/mouse per day of Vit C were referred to as the V1 group. Two groups were designated as control groups: group C had HepG2-transplanted mice without DV2 inoculation, and group NaCl had HepG2-transplanted and DV2-infected mice that were treated with a non-antioxidant 0.9% NaCl solution. DV2 was inoculated i.p. with 1×10^6^ PFU/mouse at day 10 after transplantation with HepG2 cells (5×10^6^). Immediately after DV2 inoculation, antioxidants were administered i.p. in a volume of 100 µl once a day for seven days. The mice were sacrificed on day eight p.i., and serum was isolated for the analysis of viral load, levels of antioxidants and levels of cytokines. The liver, brain, small intestine and spleen were harvested and were divided into halves. One half was homogenized and then stored at −80°C for virus titration and determination of redox status; the other half was fixed with 4% paraformaldehyde for histological examination.

### Determination of Antioxidant Levels and Lipid Peroxidation Products In Vivo

MDA levels in the serum and tissue homogenates were measured by using the thiobarbituric acid (TBA) method [21], which is based on the fact that MDA reacts with TBA to form a compound with a maximum absorbance at 532 nm. Briefly, 0.1 ml of sample was added to 0.1 ml of a solution containing 15% trichloroacetic acid, 0.375% TBA and 0.25 M hydrochloric acid. The mixture was shaken vigorously and then heated at 95°C for 40 min. The mixture was immediately cooled and centrifuged at 4,000 rpm for 10 min. Then, the absorbance of the supernatant at 532 nm was determined. The positive and negative controls included were 10 nmol/ml tetraethoxypropane and dehydrated alcohol, respectively. Results were expressed as nmol/ml of serum or nmol/mg of protein.

Total SOD (T-SOD) in the serum and tissue homogenates was determined based on the xanthine oxidase method [22]. In this method, xanthine and xanthine oxidase were incubated at 37°C for 40 min to generate superoxide radicals, which react with hydroxylamine hydrochloride to form nitrite. Then, sulfanilic acid and naphthylamine were added to react with the nitrite to form a compound with a maximum absorbance at 550 nm. Superoxide radicals are catalyzed by SOD in the samples, resulting in a reduction in the yield of nitrite. SOD activity was determined spectrophotometrically. One unit of SOD was defined as the amount of enzyme necessary to neutralize 50% of the superoxide radicals. Results were expressed as U/ml of serum or nmol/mg of protein.

CAT activity in the serum and tissue homogenates was determined by using a CAT colorimetric kit (Jiancheng Bioengineering Institute, China) according to the manufacturer’s protocol. One unit of CAT activity was defined as the number of moles of H_2_O_2_ degraded min^−1^ mg^−1^ protein. The CAT activity was expressed as U/ml of serum or U/mg of protein.

The ratio of GSSG/GSH in the liver was also determined by using a colorimetric kit (Jiancheng Bioengineering Institute, China) according to the manufacturer’s instructions. The assay was performed by using 5,5′-dithiobis-2-nitrobenzoic acid (DTNB), which reacts with GSH to form a product with a maximum absorbance at 412 nm. The GSSG level was determined by reducing GSSG into GSH by adding glutathione reductase and nicotinamide adenine dinucleotide phosphate (NADPH). The resulting GSH was reacted with DTNB. A standard curve of GSH in the liver was constructed with concentrations of GSH ranging from 0–100 µM, and the GSSG/GSH ratio was calculated by the following equation: Ratio = GSSG/(total glutathione-2GSSG).

Protein concentrations of all tissue homogenates were determined using a Nanophotometer (Implen, Germany) and were used to normalize the peroxides and antioxidant levels.

### Cytokine Quantitation

At day eight p.i., mice from each group were sacrificed and serum was isolated for cytokine expression level analysis. ELISA kits (R&D Systems, USA) were used for mouse cytokine (IL-6/TNF-α) quantitation in the serum according to the manufacturer’s protocol. Samples were analyzed in duplicate for each group.

### Distribution of DV2 Virus in the Infected Mice

Virus titers in the serum and organs were determined by using the plaque assay. The organ samples were homogenized in RPMI1640 with 2% FBS (pH 7.2) into a 50% homogenate. The suspensions were centrifuged at 12,000 rpm for 20 min at 4°C, and the supernatants were then collected and titered for viral load.

### Histopathology

Liver and brain samples were fixed with 4% paraformaldehyde and processed for paraffin sectioning and rehydration. Sections of 6 µm thickness were stained with hematoxylin and eosin (H.E.). After staining and mounting the samples, the sections were observed using a light microscope (Olympus BX61, Japan).

### Determination of ROS Production in DV2-infected HepG2 Cells

2′,7′-dichlorfluorescein-diacetate **(**DCFH-DA, Sigma) was used for ROS detection. DCFH-DA is converted to DCFH by intracellular esterases, and DCFH is further oxidized to the fluorescent product DCF by ROS [23]. The working concentration of DCFH-DA solution was 10 µM. After infecting HepG2 cells with DV2 (MOI = 1) for 12 hours and 24 hours, the HepG2 cell monolayer was treated with a DCFH-DA solution and incubated at 37°C for 20 minutes to enable the cellular uptake of DCFH-DA. Cells were washed with phosphate-buffered saline (PBS), followed by fluorescence detection using a fluorescence microscope (Olympus BX61, Japan) at an excitation wavelength of 488 nm and an emission wavelength of 525 nm.

To investigate the effect of antioxidants on ROS levels, working concentrations of antioxidants were first determined using trypan blue staining and observing the cellular morphology. Briefly, HepG2 cells were cultured in six-well plates and were incubated for 48 hours with various concentrations of the antioxidants GSH, NAC, Vit C and BSO, an inhibitor of GSH synthesis. The viability of the cells was analyzed by assessing the cellular morphology and by staining with 0.4% trypan blue. The working concentrations were as follows: 20 mM GSH, 50 µM NAC, 50 µM Vit C and 0.2 mM BSO. During the experiments, cells were treated with the antioxidants at the working concentrations during DV2 infection and were followed by DCFH-DA staining at 24 h p.i. and 48 h p.i. Mock infection was performed using DV2 that was heat-inactivated at 56°C for 30 min. ROS levels were evaluated using a fluorescence microscope.

### Effect of Antioxidants on DV2 Entry

HepG2 cells were seeded in six-well plates and infected with DV2 at an MOI of 1. Antioxidants were added to the culture media during the infection. For investigating effect of BSO on viral entry, HepG2 cells grown in six-well plates were pre-treated with 0.2 or 1 mM of BSO for 18 hr and infected with DV2 (MOI = 1). The cells were incubated at 37°C for 60 minutes to allow viral adsorption and entry in DMEM containing 2%FBS, antioxidants or BSO. Then the supernatant containing DV2 was removed, excess or unbound virus was inactivated with glycine buffer (pH 3.0) and removed by extensive washing with serum-free DMEM. The cells were freeze-thawed three times in 500 µl of serum-free DMEM to release the intracellular virus. The cell lysates were centrifuged at 12,000 rpm for 1 min at 4°C to remove the cell debris, and supernatants were collected for the detection of DV2 by plaque assay using a Vero cell monolayer under 1.3% methylcellulose overlay medium. Five independent experiments were performed for each group.

### Statistical Analysis

Statistical analysis was performed with SPSS 16.0 software. Data from separate experiments are expressed as the arithmetic mean ± standard deviation. The statistical significance of the differences between the means was determined using the Student’s t-test or one-way analysis of variance (ANOVA). Correlation between viremia and serum levels of MDA is made by Pearson correlation analysis. Significance was defined as p<0.05.

## Results

### Serum hALB Levels, Clinical Signs and Viral Distribution in HepG2-SCID Mice

After HepG2 transplantation, palpable abdominal masses developed in all mice. At five and 10 days p.t, serum hALB levels were analyzed to confirm successful transplantation. As shown in (Figure S1A), the level of hALB in the serum of all of the mice gradually increased and peaked at day 10 p.t. Gross observations revealed that masses of the implanted HepG2 cells developed in the abdominal cavity of all of the mice. The HepG2-SCID mice appeared healthy until day 50, at which point they gradually displayed illness and eventually died.

On day 10 p.t., HepG2-SCID mice were inoculated i.p. with DV2 and observed daily. After infection with DV2, the mice initially displayed ruffled fur but progressed to display anepithymia and an emaciated phenotype. The mice remained active for one week p.i. On day 10 p.i., the mice became inactive and lethargic and finally died between days 14 and 16 p.i. Unexpectedly, there were few mice that presented with paralysis of the hind-legs, although they exhibited bradykinesia and cachexia during the infection. During the biopsy examination, congestive livers and enlarged spleens or splenic atrophy were observed in some of the infected mice, but other organs such as the brain and the small intestine did not show obvious gross pathology.

On day eight p.i., serum and organ samples were harvested from DV2-infected HepG2-SCID mice for virus detection and histopathological examination. As shown in Figure S1B, the DV2 virus was detected in the serum and liver in all of the mice with titers ranging from 10^3^–10^5^ PFU/ml. The virus detection rate in the brain, spleen and small intestine was 85.7% (6/7), 71.4% (5/7) and 57.1% (4/7), respectively. Viral titers in the brain, spleen and small intestine were generally below 10^3^ PFU/ml, which was significantly lower than the viral titers in the serum and liver. In H.E. staining (as shown below), severe hemorrhage, congestion, inflammatory cell infiltration and edema were observed in the livers of infected HepG2-SCID mice, though not in uninfected HepG2-SCID mice. Slight congestion was observed in the brain and small intestine (data not shown). Immunohistochemical analysis revealed the presence of DV2 antigens in the cytoplasm of grafted HepG2 cells and within neurons in the brain (data not shown). In addition, in the infected HepG2-SCID mice, there were elevated the serum levels of TNF-α and IL-6, which were 70±30 pg/ml and 220±60 pg/ml respectively and significantly higher than that of the uninfected HepG2-SCID mice (p<0.05). These results indicate that the grafted HepG2 cells were compatible host cells for DV2 infection and replication. Thus, i.p. transplantation of HepG2 cells is a simple and useful approach for the development of a mouse model of DV2 infection.

### Changes in the Oxidative Stress Parameters in HepG2-SCID Mice After DV2 Infection

**Figure 1:**
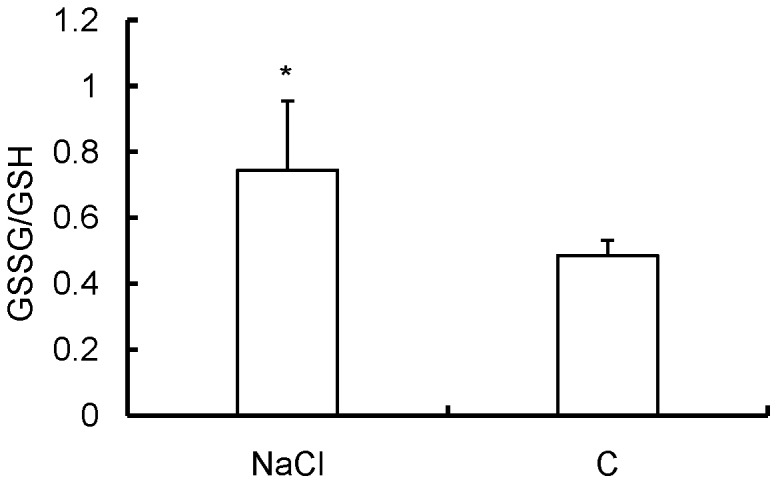
Alteration of the GSSG/GSH ratio in the liver of HepG2-SCID mice after DV2 infection. NaCl: DV2 infected mice with treatment of 0.9%NaCl; C: HepG2 transplanted mice without DV2 infection. Values shown are means ± S.D. from seven mice (*p<0.05 vs. C group).

To observe whether DV2 infection can induce oxidative stress in mice, the levels of T-SOD, CAT, GSSG/GSH ratio and MDA were measured as an indication of the redox status in the mice after DV2 infection. As shown in Table 1, T-SOD activity in the serum and liver was dramatically and significantly decreased after DV2 infection when compared to uninfected HepG2-SCID mice (p<0.01). T-SOD levels in the brain and spleen only showed a slight decrease that did not reach statistical significance. MDA levels in the serum and organs of the DV2-infected mice were significantly elevated when compared to the controls (p<0.05). CAT activity in the liver showed a significant decrease when compared with the controls (p<0.05), whereas there was no obvious change in CAT levels in the serum and other organs. Notably, when compared with other organs, only the liver showed a significant alteration in all of the redox status indicators analyzed, which prompted us to determine the ratio of GSSG to GSH in the liver. As expected, the GSSG/GSH ratio showed a marked decrease after DV2 infection (Figure 1). In combination with the above histopathological examination, these results indicate that DV2 infection can induce oxidative stress and that the liver appears to be the major target organ for oxidative injury during DV2 infection.

**Table 1:** Alterations of T-SOD, MDA and CAT in the serum and major organs of HepG2- SCID mice after DV2 infection.

			T-SOD (U/ml.mg)	MDA (nmol/ml.mg)	CAT (U/ml.mg)
Serum		Inf^a^	232.40±36.52**	5.37±1.44*	2.04±0.67
		Cont^b^	315.96±41.43	4.02±0.36	2.48±1.11
Liver		Inf	265.40±67.18*	6.23±0.28*	7.09±2.30*
		Cont	367.88±39.23	5.16±0.80	10.32±0.58
Brain		Inf	110.11±41.09	0.62±0.13*	17.54±4.97
		Cont	148.87±55.27	0.35±0.16	21.26±9.02
Spleen		Inf	62.76±10.49	1.73±0.43*	17.50±4.35
		Cont	66.85±10.06	0.98±0.23	16.96±4.41

Mice were infected i.p. with 10^6^ PFU of DV2 (Tr1751), and were sacrificed at day 8 p.i. Samples from serum and major organs were prepared for determination of total superoxide dismutase (T-SOD), malondialdehyde (MDA) and catalase (CAT). Values are means ± S.D. of seven mice each group. (*p<0.05, **p<0.01).

a Inf, HepG2-SCID mouse with DV2 infection i.p.

b Cont, control group (HepG2-SCID mice without infection).

**Figure 2:**
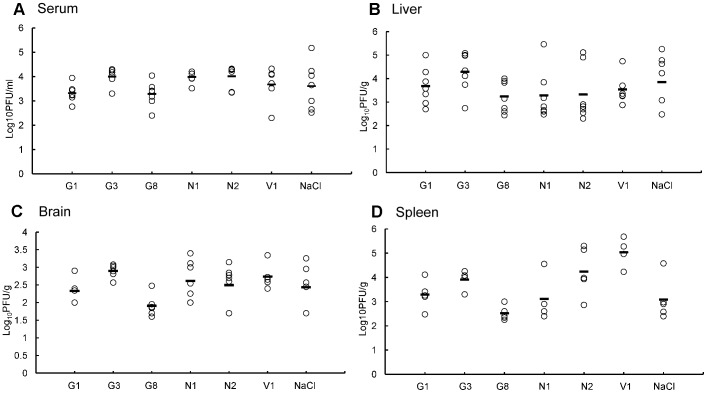
Virus titers in the serum and organs in the mice with or without antioxidant administration. The mice were infected i.p. with 10^6^ PFU of DV2 and were sacrificed at day 8 p.i. Virus titers from the infected mice with or without antioxidant administration were determined for the serum (A), liver (B), brain (C) and spleen (D). G1, G3 or G8: DV2 infected mouse with Glutathione (GSH) treatment of 1 mg/day or 3 mg/day or 8 mg/day; N1 or N2: DV2 infected mouse with *N*-acetyl-l-cysteine (NAC) treatment of 1 mg/day or 2 mg/day; V1: DV2 infected mouse with Vitamin C (VitC) treatment of 1 mg/day; NaCl: DV2 infected mice with treatment of 0.9%NaCl. Results are expressed as log_10_ PFU per milliliter of serum and per gram of the organs.

**Figure 3:**
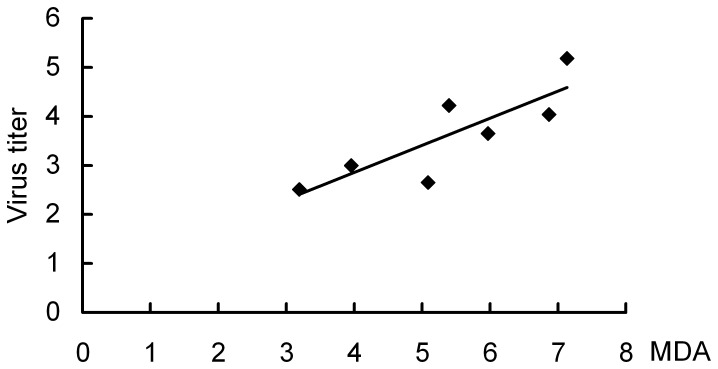
Correlation between viremia and serum levels of MDA in HepG2- SCID mice after DV2 infection. The analysis showed a positive correlation between two parameters (correlation = 0.838, R2 = 0.4293, p = 0.019). Y and X axes represent virus titer (viremia, Log_10_PFU/ml) and MDA (U/ml) levels respectively.

In addition, DV2 was detected in the serum of all of mice with titers ranged from 2.6–5.2 Log10PFU/ml (Figure 2A). The correlation between viremia and MDA level was analyzed and they showed a positive correlation (correlation = 0.838, p = 0.019, Figure 3), indicating a common relationship between virus infection and oxidative stress, and further implying antioxidant may be an available strategy for treatment of the disease. Moreover, correlations between DV2 titers and CAT or T-SOD activity in the liver or serum were also analyzed. However, there are no correlations between DV2 titers and CAT or T-SOD activity (data not shown).

### Effect of Antioxidants on the Progression of DV2 Infection in HepG2-SCID Mice

To test whether antioxidants have a protective effect on oxidative stress induced by DV2 infection in vivo, GSH, NAC and Vit C were administered once a day following DV2 infection. On day eight p.i., serum and organ samples were collected for the determination of viral load as well as the above parameters (MDA, SOD, CAT and GSSG/GSH ratio) and histopathological examination. As shown in Figure 2, although the virus detection rate was rarely affected by treatment with most of the antioxidants at the doses used in this study, the DV2 load in the serum and organs was decreased in the G8 group of mice (GSH at 8 mg/mouse per day). In addition, NAC at doses of 1 and 2 mg/mouse per day, which is a precursor of GSH, showed inhibitory activity as indicated by DV2 titers in the liver when compared with the NaCl group. These findings indicate that both GSH and NAC can inhibit DV2 replication in the liver.

**Figure 4:**
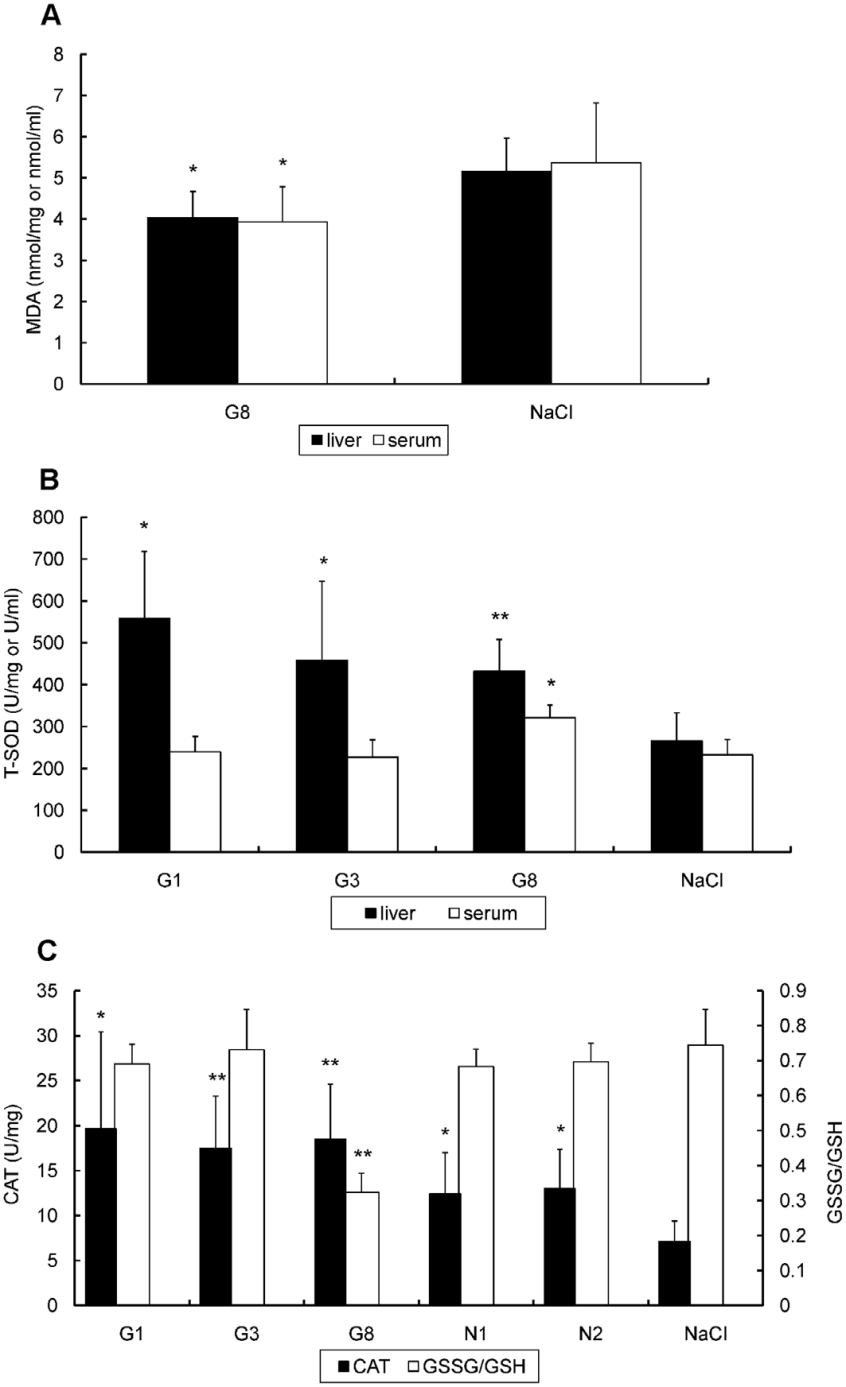
Alteration of the oxidative stress parameters of DV2-infected HepG2-SCID mice with or without antioxidant administration. A: malondialdehyde (MDA) levels in the liver and serum. B: total superoxide dismutase (T-SOD) activity in the liver and serum. C: catalase (CAT) activities (Y-axis on the left) and GSSG/GSH ratio (Y-axis on the right) in the liver. G1, G3 or G8: DV2 infectedmouse with Glutathione (GSH) treatment of 1 mg/day or 3 mg/day or G8; N1 or N2: DV2 infected mouse with *N*-acetyl-l-cysteine (NAC) treatment of 1 mg/day or 2 mg/day; NaCl: DV2 infected mice with treatment of 0.9%NaCl. Values shown are means ± S.D. of seven mice from each group. (*p<0.05, **p<0.01 v.s. NaCl group).

To determine whether antioxidant treatment can affect DV2-induced oxidative stress, the levels of MDA, GSSG/GSH, T-SOD and CAT were measured in the serum and/or liver of DV2-infected mice with or without antioxidant treatment. As mentioned above, DV2 infection appears to induce oxidative stress and liver injury in infected SCID-HepG2 mice (Table 1). After the administration of the various antioxidants, the level of MDA decreased in the serum and liver in the G8 group (Figure 4A), which was significantly different from that of the NaCl group (p<0.05). Simultaneously, a high dose of GSH (8 mg/mouse per day) noticeably improved T-SOD activity in both the serum and liver, while low doses (1 and 3 mg/mouse per day) of GSH increased T-SOD activity only in the liver (Figure 4B). Interestingly, increased CAT levels in the liver were not only detected in mice treated with GSH at all of the doses used but also in the N1 and N2 groups of mice. A decrease in the GSSG/GSH ratio in the liver was also consistently observed in the G8 group (Figure 4C).

**Figure 5:**
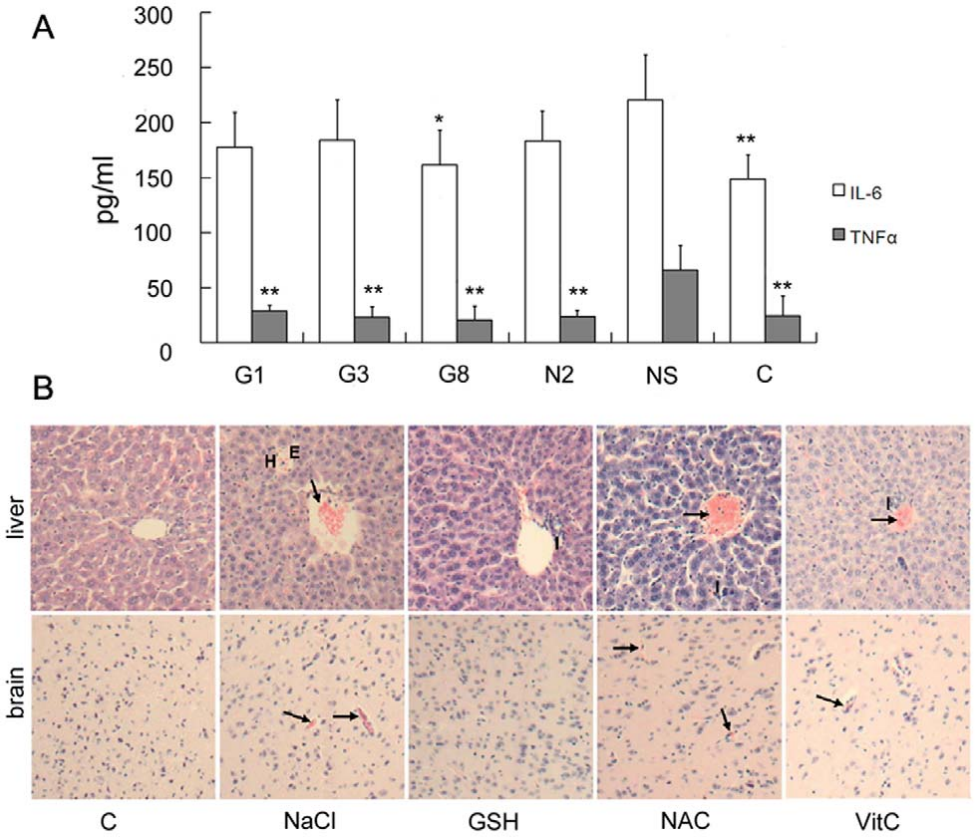
Changes of serum cytokine levels and histopathological observation in DV2 infected HepG2-SCID mice after antioxidant administration. A: serum levels of tumor necrosis factor-α (TNF-α) and interlukin-6 (IL-6) in the mice with or without antioxidant administration. G1, G3 or G8: DV2 infected mouse with Glutathione (GSH) treatment of 1 mg/day or 3 mg/day or 8 mg/day; N2: DV2 infected mouse with *N*-acetyl-l-cysteine (NAC) treatment of 2 mg/day; NaCl: DV2 infected mice with treatment of 0.9%NaCl; C: HepG2 transplanted mice without DV2 infection. Values shown are means ± S.D. (n = 7). (*p<0.05, **p<0.01 vs. NaCl group). B: Histopathological examination of the liver and brain in DV2-infected HepG2-SCID mice with or without antioxidant administration. Uninfected HepG2-SCID mice were used as controls (C). DV2-infected HepG2-SCID mice were administered with 0.9% NaCl, GSH (8 mg/day per mouse), NAC (1 mg/day per mouse) or Vitamin C (Vit C, 1 mg/day per mouse) for seven days. C: HepG2 transplanted mice without DV2 infection. Liver sections showed hemorrhage (H), inflammatory cell infiltration (I), edema (E) and severe congestion (arrows) in the NaCl, NAC and Vit C treatment groups, whereas GSH treatment ameliorated the above histopathological changes. Similarly, brain sections of the NaCl, NAC, and Vit C groups showed slight congestion (arrows), and there was no obvious change in the GSH group. H.E. staining, ×200 magnification.

As compared with significantly elevated the serum levels of TNF-α and IL-6 seen in the infected HepG2-SCID mice, reduced serum levels of TNF-α were observed after treatment with GSH at all of the doses tested or after treatment with NAC at 2 mg/mouse per day (Figure 5A); these levels approached those of the uninfected HepG2-SCID mice (group C). But reduced serum IL-6 levels were only seen in G8 group (GSH 8 mg/mouse per day). These results suggest that GSH can inhibit the production of TNF-α and IL-6, resulting in the inhibition of oxidative injury while NAC treatment has a limited effect on IL-6 production.

Histopathological examination was performed on the infected HepG2-SCID mice with or without the administration of antioxidants. As shown in Figure 5B, there were no obvious pathological changes in the liver and brain of the uninfected HepG2-SCID mice. However, severe hemorrhage, marked congestion, inflammatory cell infiltration and edema in the liver and slight congestion in the brain were observed in DV2-infected HepG2-SCID mice. Treatment with GSH at 8 mg/mouse per day ameliorated DV2-induced liver injury, and only mild congestion and inflammatory cell infiltration were observed in the liver. Furthermore, the brain occasionally showed slight congestion after the administration of high doses of GSH. However, except for GSH, the other antioxidants used in this study showed only modest prevention of liver injury. These results indicate that GSH has a significant inhibitory effect on DV2-induced oxidative stress and can inhibit liver damage during DV2 infection.

### ROS Production in HepG2 Cells after DV2 Infection and the Inhibitory Effects of the Antioxidants on this Process

**Figure 6:**
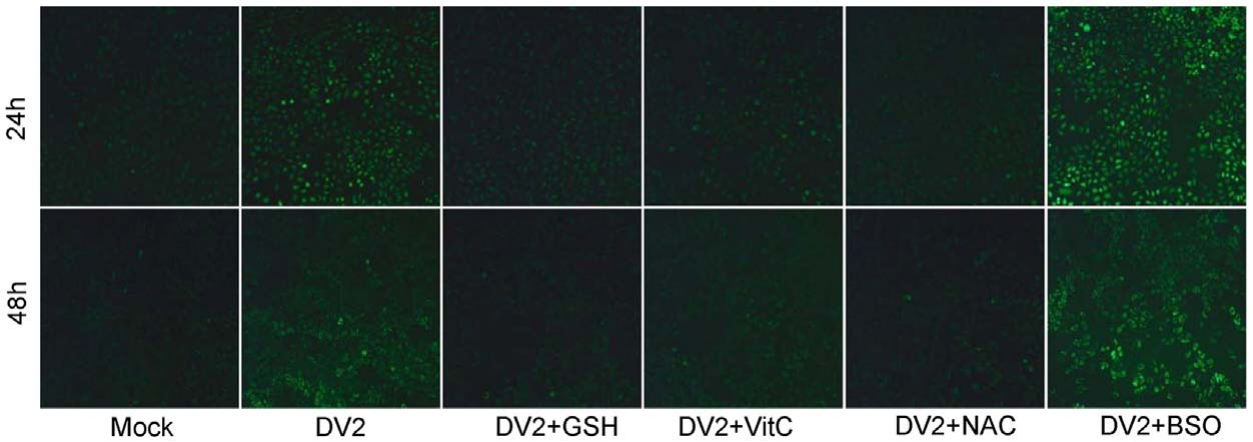
Reactive oxygen species (ROS) production in DV2-infected HepG2 cells with antioxidant or buthionine sulfoximine (BSO) administration. Mock: HepG2 cells infected with heat-inactivated (56°C for 30 min) DV2, DV2: DV2 infected HepG2 cells at 1 of multiplicity of infection (MOI), DV2+GSH: DV2 infected HepG2 cells with Glutathione (GSH) treatment of 20 mM, DV2+VitC: DV2 infected HepG2 cells with Vitamin C (VitC) treatment of 50 µM, DV2+NAC: DV2 infected HepG2 cells with *N*-acetyl-l-cysteine (NAC) treatment of 50 µM, DV2+BSO: DV2 infected HepG2 cells with BSO treatment of 0.2 mM. A significant increase in the fluorescence intensity was observed in HepG2 cells at 24 h and 48 h after DV2 infection. DV2-infected HepG2 cells treated with antioxidants or BSO altered ROS levels. ×100 magnification.

To explore whether DV2 infection induces oxidative stress in vitro, production of ROS in cultured HepG2 cells was determined by using the DCFH-DA fluorescence method. Compared with mock infected cells, a significant increase in the fluorescence intensity, which reflects the level of ROS production, was observed in DV2-infected HepG2 cells at 24 and 48 h after DV2 infection (Figure 6), indicating that DV2-induced oxidative stress also occurs in vitro. Treatment with three antioxidants including GSH,NAC and Vit C showed an inhibitory effect on ROS production at 24 and 48 h, whereas 0.2 mM of BSO enhanced ROS production after DV2 infection.

### Effects of Antioxidants on Viral Entry into HepG2 Cells

**Figure 7:**
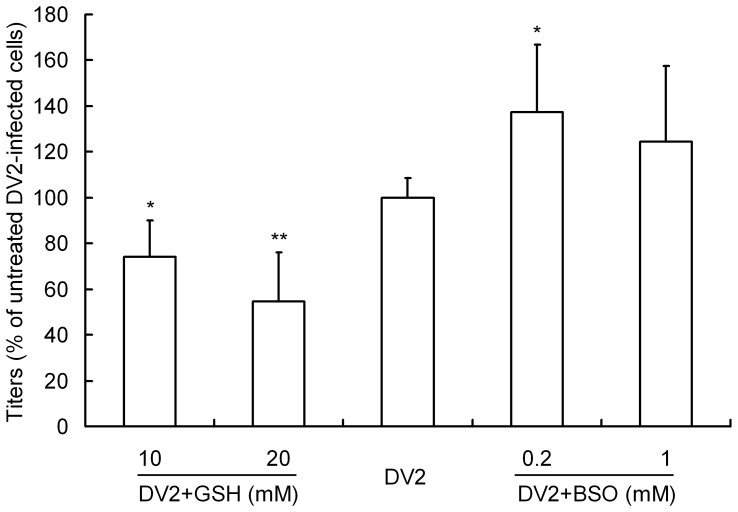
Effect of antioxidants and buthionine sulfoximine (BSO) on the entry of DV2 into HepG2 cells. Confluent monolayers of HepG2 cells were infected with DV2 at 1 of multiplicity of infection (MOI). Medium containing Glutathione (GSH) or BSO was incubated with HepG2 cells during the virus adsorption period. Then, the cells were washed three times and freeze-thawed to release intracellular virus. Virus titers were detected by using the plaque assay and were expressed as a percentage of the DV2-infected group that did not receive treatment. DV2: DV2 infected HepG2 cells (MOI = 1), DV2+GSH: DV2 infected HepG2 cells with GSH treatment of 10 mM or 20 mM, DV2+BSO: DV2 infected HepG2 cells with BSO treatment of 0.2 mM or 1 mM. Values shown are means ± S.D. from five independent experiments. (**p<0.01 v.s. DV2 group).

To investigate the inhibitory effect of the antioxidants on DV2 entry, 10 mM or 20 mM GSH, 50 µM NAC, 50 µM Vit C and 0.2 mM or 1 mM BSO were added to the culture media. As shown in Figure 7, 10 mM or 20 mM GSH inhibited the entry of DV2 into HepG2 cells by approximately 30 or 50%, whereas BSO markedly enhanced the entry of DV2 into HepG2 cells by more than 40%. However, NAC and Vit C did not have any obvious effect on viral entry (data not shown). These results indicate that exogenous GSH can significantly inhibit the entry of DV2 into host cells with dose-dependent pattern.

## Discussion

### Intraperitoneal Injection of HepG2 Cells into SCID Mice is a Viable Approach for Developing a Successful Mouse Model for DV Infection

DV infection is a rapidly growing health problem, with an estimated 2.5 billion people at risk and an estimated 50 million annual dengue infections worldwide [24]. However, the pathogenesis of human dengue infection remains unclear, and no successful vaccine is currently available. Previous studies have suggested that ROS-induced oxidative stress as part of the host cell response to viral infections might play an important role in the pathogenesis of a variety of viral infections, including DV infection.

In this study, to analyze whether antioxidants have inhibitory effects on the oxidative stress induced by DV infection in vivo, our previously developed mouse model was used with a slight modification that used i.p. injection of HepG2 cells into SCID mice rather than splenic injection [20]. After HepG2 cell transplantation, a gradual increase in the serum levels of hALB was detected in all of the mice, indicating a 100% success rate in transplantation. In addition, the mice appeared healthy and survived for approximately 50 days p.t. These results suggest that i.p. transplantation of HepG2 cells is a simple and effective method for xenograft generation.

After inoculation with DV2, our model not only partially replicated the human clinical manifestations of DV2 infection but also showed high viremia with a 100% detection rate of DV2 after infection. Significant viral titers were also detected in major organs observed. Immunohistochemistry staining showed the presence of virus in transplanted HepG2 cells and neurons (data not shown). In recent studies, Prestwood and Rice et al [40]–[41] demonstrated that macrophage populations, initially in the spleen and other lymphoid tissues, and later in non-lymphoid tissues including gut-associated tissues and liver, are major targets of DV infection in AG129 mice. In combination with above reports, those indicated a possibility that DV2 could replicate in macrophage populations and other cells, except for grafted HepG2 cells, in our mouse model. More importantly, there were few mice that showed paralysis of the hind legs during disease progression when compared with our previous model, in which hind limb paralysis was observed in most of the SCID mice that received HepG2 xenografts by the splenic route. This difference suggests that the current model more closely mimics human DV infection because paralysis is not a clinical sign for human DV disease [25]–[27]. Therefore, our results suggest that i.p. injection of HepG2 cells into SCID mice is an effective approach for developing a successful mouse model for DV infection, and our mouse model is useful for studying pathogenesis of DV infection.

### Oxidative Stress and Liver Injury Induced by DV2 Infection can be Ameliorated by Treatment with GSH, but not NAC

Oxidative stress has been implicated in the pathogenesis of several viral infectious diseases. ROS generation, due to the activation of inflammatory cells such as neutrophils within the organs during disease progression, is considered to be an important host antiviral defense and is necessary for systemic viral clearance. In the present study, the oxidative stress status was examined in the HepG2-SCID mice infected with DV. Increased levels of MDA in the serum and organs and decreased serum T-SOD activity as well as enhanced serum levels of TNF-α and IL-6 were observed after DV2 infection. Especially, a positive correlation between viremia and levels of MDA was found, further indicating close relationship between DV2 infection and oxidative stress (Figure 3). Notably, significant reductions of T-SOD and CAT activities, as well as an elevated MDA level and GSSG/GSH ratio, were detected in the liver. In addition, HepG2 cells infected with DV2 also showed enhancement of ROS production. These results suggest that DV2 infection can cause oxidative stress both in vivo and in vitro. Histopathological examination revealed significant pathology including hemorrhage, congestion and edema in the liver, whereas other organs showed only moderate histopathological changes. Taken together, these results further suggest that DV2 infection induces oxidative stress and that the liver might be a major target organ of oxidative injury induced by DV2 infection.

Our findings are supported by several previous studies that have demonstrated that an increase in the concentration of lipid peroxidation markers such as hydroperoxides, MDA and 4-hydroxyalkenals in the serum of dengue patients and high aminotransferase levels were observed in over 60% of dengue patients [17]. Moreover, hepatic damage including steatosis, swelling and necrosis was often observed in dengue patients as well as BALB/c mice intraperitoneally inoculated with a DV2 isolate [1], [28]. Furthermore, increased oxidative load and decreased AOEs activity have also been reported in other viral infections such as HIV [29], hepatitis C [30] and influenza [31], [32]. In combination with those reports, our results demonstrate that oxidative stress is not only a common consequence of viral infections including DV infection but also an important cause of the liver dysfunction and injury observed in dengue patients.

The mechanism of oxidative stress induced by DV infection is unclear; however, the mechanism might involve robust ROS production and antioxidant consumption elicited by viral replication [19], which results in cellular damage and organ pathology. Thus, antioxidants are potentially effective strategies against viral infection and viral infection-associated symptoms. Therefore, the antioxidant effects of GSH, NAC and Vit C were further investigated in this study.

GSH contains a thiol group (-SH) and is the major endogenous antioxidant produced by cells. It is capable of preventing damage to important cellular components by directly neutralizing ROS with reducing disulfide bonds and maintaining exogenous antioxidants such as vitamin C and E in their reduced forms [33]. In addition, GSH affects the function of the immune system and regulates cytokine production. Because it is essential to proper liver function, GSH has been widely used clinically for the inhibition of liver inflammation. In previous study, we found that DV2 infection resulted in a decrease in intracellular GSH levels, which caused NF-κB activation and enhanced DV2 production. Supplemental GSH significantly inhibited the activation of NF-κB and decreased the production of DV2 in HepG2 cells [19]. Accordingly, in the current study, we observed an obvious improvement in the cellular redox status, including marked decreases in the levels of MDA, the GSSG/GSH ratio and TNF-α and IL-6 levels as well as significantly elevated T-SOD and CAT activities in the serum and/or liver and a reduction in liver damage in the G8 group (GSH 8 mg/mouse per day). However, GSH administered at low doses showed only minor effects on the above parameters. Notably, the G8 group also showed a reduced viral load in the organs, especially in the liver and brain. These results indicate that high doses of GSH have a significant inhibitory effect on oxidative stress, liver injury and virus production. Furthermore, high dose of GSH appears to be a potentially useful therapeutic agent for improving disease conditions associated with DV infection.

Our results are in partial agreement with other antiviral reports in which GSH and its analogs were used for testing the antiviral effects on several viral infections. Treatment with GSH showed an inhibitory effect on the production of influenza A virus or HSV-1 both in vitro and in vivo [14], [7]. Pretreatment with exogenous H_2_S or GSH could suppress rhinovirus-induced superoxide radical production and reverse intracellular GSH levels in vitro [34]. GSH administered intramuscularly caused a reduction in the weights of the spleen and lymph node in HIV-infected mice [15]. Taken together, these data suggest that the inhibition of oxidative pathways by antioxidants such as GSH has therapeutic potential for related clinical diseases.

NAC treatment conferred an inhibitory effect on DV titers in the liver. The results on NAC treatment are partially consistent with most previous studies that have found that treatment with NAC results in marked antiviral activity. A previous study has shown that NAC has an inhibitory role in the replication of the highly pathogenic H5N1 influenza A virus and the H5N1-induced expression of pro-inflammatory molecules in the A549 cell line [35]. Oral administration of NAC can cause an increase in the whole blood GSH levels in HIV infection [36], reduce viral replication in stimulated CD4 lymphocytes and re-establish immunological reactivity to the HIV virus in AIDS patients [37]. Moreover, treatment with NAC (1 g/kg per day orally) significantly decreased the mortality in mice infected with the influenza A virus [38]. However, NAC administration only increased the CAT activity and reduced the DV2 titers in the liver and inhibited the serum levels of TNF-α and ROS production in vitro, indicating that NAC only has a certain degree of protection on oxidative stress in this study as compared with GSH. Although other unapparent explanations may exist, we suspect that the lack of a protective effect by NAC is associated with the doses used. Increasing the NAC dose might result in a degree of protection similar to that of GSH. In our ongoing study, we are investigating whether NAC at different doses can inhibit oxidative stress induced by DV infection.

### GSH can Provide Protection by Increasing the Intracellular Reducing Form and Inhibiting Viral Replication

Although it is difficult to determine the precise mechanisms by which GSH provides protection during DV2 infection, we suspect that several factors are involved in this process, including the detoxification of ROS, the inhibition of the activation of NF-κB and reduction of the levels of inflammatory cytokines. GSH is known to regulate a variety of cellular functions by redox-dependent mechanisms. Decreased GSH levels and increased levels of lipid peroxidation could activate NF-κB, which results in the secretion of inflammatory mediators [39] and may contribute to tissue damage and the symptoms of DHF/DSS. In the current study, increased oxidative stress and increased serum levels of TNF-α and IL-6 were observed after DV2 infection. Treatment of DV2-infected mice with GSH ameliorated the redox state by increasing the activity of AOEs such as SOD and CAT and decreasing the production of MDA and inflammatory cytokines such as TNF-α. In addition, in vitro analysis of DV2 infection of HepG2 cells showed that three antioxidants used in this study could inhibit ROS production induced by DV2 infection, whereas BSO promoted ROS production in DV2-infected HepG2 cells. TNF-α and IL-6 are early cytokine mediators of inflammatory signaling and effectively amplify the host inflammatory response to viral infection. Our previous study has shown that the addition of exogenous GSH can inhibit the activation of NF-κB, whereas BSO enhances NF-κB activity. Collectively, these results suggest that oxidative stress induced by DV2 infection not only causes direct tissue damage but also stimulates the host inflammatory responses via the activation of the NF-κB pathway. GSH can ameliorate tissue damage by providing reducing disulfide bonds and increasing the activity of SOD and CAT, which scavenge ROS and maintain the redox status of the host cell. Therefore, the oxidative stress-associated protective effect of GSH is due to the reduction in the secretion of TNF-α and IL-6 by inhibiting lipid peroxidation products and activating NF-κB, although other important mediators might be affected by GSH treatment.

Our previous study demonstrated that exogenous GSH can inhibit DV2 production in HepG2 cells [19]. In this study, we found that treatment with GSH inhibited DV2 entry into HepG2 cells, whereas BSO enhanced DV2 entry into HepG2 cells. NAC and Vit C did not appreciably affect viral entry. GSH treatment also decreased the DV2 load in all of the organs analyzed. There is a close association between GSH levels and DV2 infection, and the modulation of the intracellular redox environment is essential for the initiation and maintenance of viral replication [19]. We found that GSH can inhibit the DV replication cycle by targeting entry and propagation via the alteration of the intracellular reducing environment. Taken together, our results indicate that protection by GSH is closely related to the balance of oxidant and antioxidant status, the inhibition of viral replication and the reduction of the DV2-mediated inflammatory reaction and liver injury induced by oxidative stress. Our results suggest that GSH might provide an effective strategy for the prevention and treatment of DV infection.

In summary, our study demonstrates that DV2 infection results in a significant alteration in the oxidative status in the liver, as shown by increased ROS production, MDA levels and the GSSG/GSH ratio and decreased levels of SOD and CAT. Moreover, DV2 infection induces the expression of TNF-α and IL-6 in the serum of the mice. We have shown that treatment with exogenous GSH reverses all of these aberrant parameters and inhibits viral entry and viral load in major organs. According to our results and those of others, we speculate that the mechanisms involved in the GSH-associated protective effects during DV2 infection are related to the ability of GSH to balance the oxidant and antioxidant ratio by scavenging free radicals, reducing the secretion of inflammatory mediators and inhibiting DV2 replication. GSH is an abundant natural antioxidant and has no known toxicity. Therefore, our results make a strong case for the use of GSH as a therapeutic agent for the prevention of liver damage elicited by lipid peroxidation during DV infection.

## Supporting Information

Figure S1 **Serum levels of human albumin** (**hALB**) **and viral distribution in HepG2-SCID mice with or without DV2-infection.** A: hALB levels in the serum of mice at 5 and 10 days after HepG2 cell transplantation. Values shown are means ± S.D. (n = 10) B: Distribution of DV2 in HepG2-SCID mice. Results are expressed as log_10_ PFU per milliliter of serum or per gram of organ (n = 7). Each point represents an individual mouse.
(DOC)
