# Supplementary material for: Inhibitory Effect of Glutathione on Oxidative Liver Injury Induced by Dengue Virus Serotype 2 Infections in Mice
Source: PLoS One. 2013 Jan 30;8(1):e55407. doi: 10.1371/journal.pone.0055407 (PMC3559498; doi:10.1371/journal.pone.0055407)
Supplement: Figure S1 — Serum levels of human albumin (hALB) and viral distribution in HepG2-SCID mice with or without DV2-infection. A: hALB levels in the serum of mice at 5 and 10 days after HepG2 cell transplantation. Values shown are means ± S.D. (n = 10) B: Distribution of DV2 in HepG2-SCID mice. Results are expressed as log10 PFU per milliliter of serum or per gram of organ (n = 7). Each point represents an individual mouse. (DOC) [file pone.0055407.s001.doc]

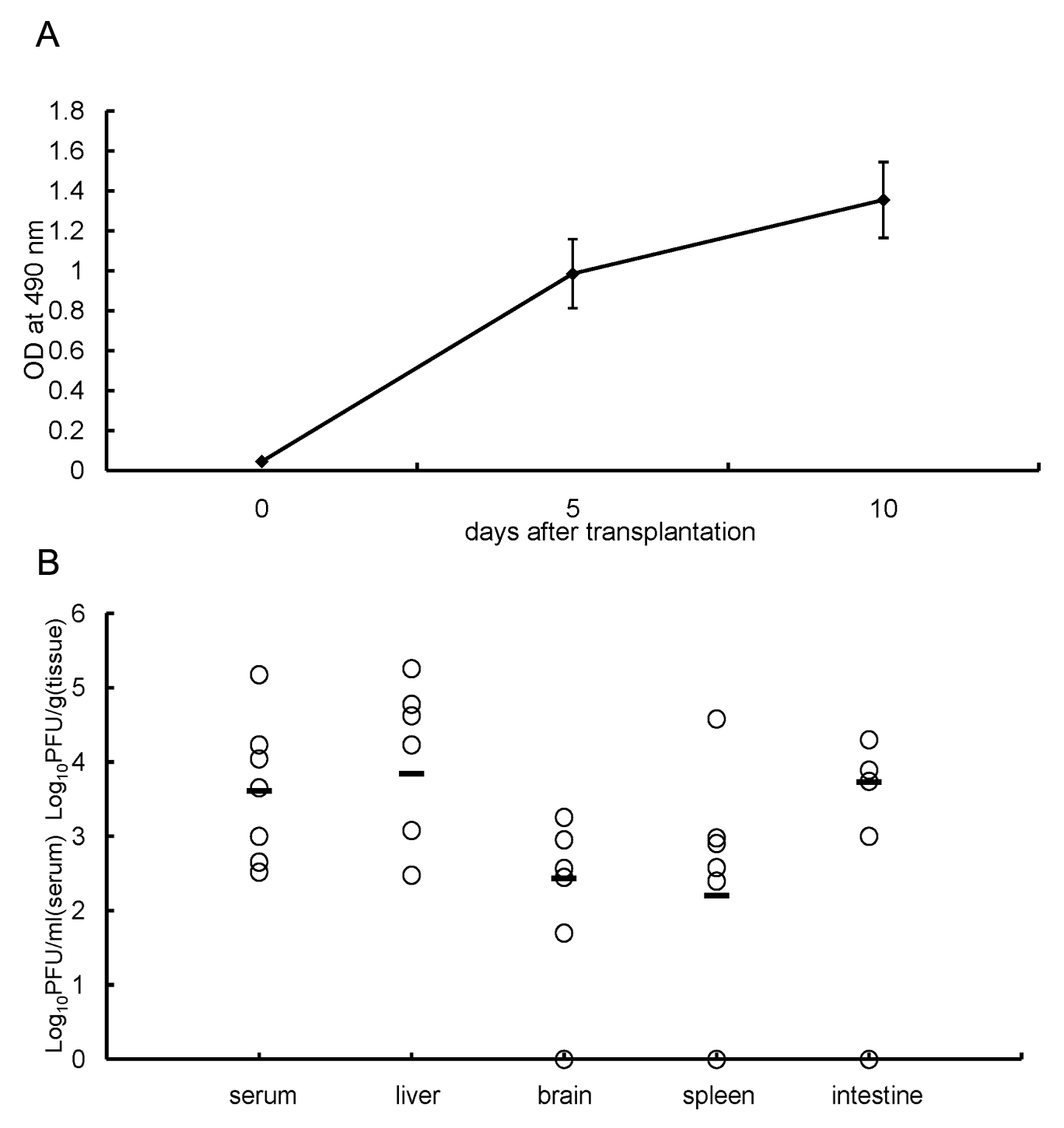


Figure S1. Serum levels of human albumin (hALB) and viral distribution in HepG2-SCID mice with or without DV2-infection. A: hALB levels in the serum of mice at 5 and 10 days after HepG2 cell transplantation. Values shown are means ± S.D. (n=10) B: Distribution of DV2 in HepG2-SCID mice. Results are expressed as log10 PFU per milliliter of serum or per gram of organ (n=7). Each point represents an individual mouse.
